# Supplementary material for: Electronic data review, client reminders, and expanded clinic hours for improving cervical cancer screening rates after the COVID-19 pandemic shutdowns: A multicomponent quality improvement program
Source: eLife. 2023 Aug 22;12:e85724. doi: 10.7554/eLife.85724 (PMC10471159; doi:10.7554/eLife.85724)
Supplement: Figure 1—source data 1. [file elife-85724-fig1-data1.pdf]

Pap Clinic Patient Satisfaction Survey

Patient Survey - English
PAP Clinic Encounters

Thank you again for completing your last Pap screening with East Boston Neighborhood Health Center. To help us continually improve, please answer a few short questions about your experience. Your responses will be processed by a third-party survey research firm with results combined and reported with those of other respondents.

1: How helpful was it to you that the center reached out to you to offer a Pap test?

❑ Very Helpful ❑ Somewhat Helpful ❑ Not Helpful

2: Do you think that you would have scheduled a Pap test in the next 6 months if the center had not reached out to you to schedule?

❑ Yes ❑ No ❑ Don't Remember/Not Sure

3: When you were contacted by phone, how would you rate the courtesy and helpfulness of the phone attendant who scheduled your test?

❑ Poor ❑ Fair ❑ Good ❑ Excellent ❑ NA

4: How would you rate the convenience of the day and time that you scheduled the test?

❑ Poor ❑ Fair ❑ Good ❑ Excellent ❑ NA

5: About how long did the entire test take from the time you arrived until you left?

❑ Less than 30 min
❑ Over 30 min - 45 min
❑ Over 45 min - 1 hour
❑ Over 1 hour - 2 hours
❑ Over 2 hours
❑ Don't Remember/Not Sure

5a: How would you rate your satisfaction with this duration of ____for your Pap test?

❑ Poor ❑ Fair ❑ Good ❑ Excellent ❑ NA

6: How would you rate the courtesy and helpfulness of the Medical Assistant who helped the provider administer the Pap test?

❑ Poor ❑ Fair ❑ Good ❑ Excellent ❑ NA

7: How would you rate the courtesy and helpfulness of the Provider who administered the Pap test?

❑ Poor ❑ Fair ❑ Good ❑ Excellent ❑ NA

8: How would you rate the overall quality of how the Pap test procedure was administered?

❑ Poor ❑ Fair ❑ Good ❑ Excellent ❑ NA

9: Were all of your expectations met during the Pap test?

❑ Yes ❑ No ❑ Don't Remember/Not Sure

[If No] Please describe how the center could have better met your expectations during this visit:

___

___

___

10: How likely will you be to schedule another Pap test with East Boston Neighborhood Health Center in the future when you receive a reminder?

❑ Very Likely ❑ Somewhat Likely ❑ Not Likely ❑ Not Sure (Live out-of-town)

11: How would you rate your overall satisfaction with your Pap test experience?

❑ Poor ❑ Fair ❑ Good ❑ Excellent ❑ NA

12: Do you have any comments or suggestions related to this experience?

____

____

____

____

Would you like for someone from the center to contact you to discuss any concerns expressed in this survey?

❑ Yes ❑ No

©2021. The Crossroads Group, Inc.

Measurable Improvement Solutions
